# Suggested mechanisms for Zika virus causing microcephaly: what do the genomes tell us?

**DOI:** 10.1186/s12859-017-1894-3

**Published:** 2017-12-28

**Authors:** Se-Ran Jun, Trudy M. Wassenaar, Visanu Wanchai, Preecha Patumcharoenpol, Intawat Nookaew, David W. Ussery

**Affiliations:** 1 Department of Biomedical Informatics, University of Arkansas for Medical Sciences, Little Rock, AR USA; 2 Molecular Microbiology and Genomics Consultants, Zotzenheim, Germany

**Keywords:** Zika virus, Comparative genomics, Positive selection, Phosphorylation, Microcephaly

## Abstract

**Background:**

Zika virus (ZIKV) is an emerging human pathogen. Since its arrival in the Western hemisphere, from Africa via Asia, it has become a serious threat to pregnant women, causing microcephaly and other neuropathies in developing fetuses. The mechanisms behind these teratogenic effects are unknown, although epidemiological evidence suggests that microcephaly is not associated with the original, African lineage of ZIKV. The sequences of 196 published ZIKV genomes were used to assess whether recently proposed mechanistic explanations for microcephaly are supported by molecular level changes that may have increased its virulence since the virus left Africa. For this we performed phylogenetic, recombination, adaptive evolution and tetramer frequency analyses, and compared protein sequences for the presence of protease cleavage sites, Pfam domains, glycosylation sites, signal peptides, trans-membrane protein domains, and phosphorylation sites.

**Results:**

Recombination events within or between Asian and Brazilian lineages were not observed, and likewise there were no differences in protease cleavage, glycosylation sites, signal peptides or trans-membrane domains between African and Brazilian strains. The frequency of Retinoic Acid Response Element (RARE) sequences was increased in Brazilian strains. Genetic adaptation was also apparent by tetramer signatures that had undergone major changes in the past but has stabilized in the Brazilian lineage despite subsequent geographic spread, suggesting the viral population presently propagates in the same host species in various regions. Evidence for selection pressure was recognized for several amino acid sites in the Brazilian lineage compared to the African lineage, mainly in nonstructural proteins, especially protein NS4B. A number of these positively selected mutations resulted in an increased potential to be phosphorylated in the Brazilian lineage compared to the African linage, which may have increased their potential to interfere with neural fetal development.

**Conclusions:**

ZIKV seems to have adapted to a limited number of hosts, including humans, during which its virulence increased. Its protein NS4B, together with NS4A, has recently been shown to inhibit Akt-mTOR signaling in human fetal neural stem cells, a key pathway for brain development. We hypothesize that positive selection of novel phosphorylation sites in the protein NS4B of the Brazilian lineage could interfere with phosphorylation of Akt and mTOR, impairing Akt-mTOR signaling and this may result in an increased risk for developmental neuropathies.

**Electronic supplementary material:**

The online version of this article (10.1186/s12859-017-1894-3) contains supplementary material, which is available to authorized users.

## Background

The Zika virus (ZIKV) pandemic that has spread out of Brazil recently has become a serious threat to human health. Although this viral vector-born disease was originally considered an African sylvatic zoonosis that caused relatively mild symptoms only, it is now evident that it can result in serious complications, such as neuropathies and teratogenic damage to the developing fetus. Post-infectious sequela such as Guillain-Barré syndrome (GBS) are most likely caused by auto-immune responses of the host, resulting from cross-reacting antibodies, similar to post-infectious GBS that can occur during or following infection of other viral pathogens (e.g. Dengue and Influenza virus [1, 2]). In contrast, the teratogenic effects that have emerged during the recent ZIKV outbreak in South America, with the majority of cases reported from Brazil, are most likely the result of the virus reaching the developing fetus, and infecting its brain tissue [3, 4]. Despite high exposure to ZIKV in Africa in the past, resulting in high seropositive rates (e.g. 30% in Nigeria [5]), birth defects have never been associated with ZIKV infection in this continent. Something has changed, and the genetic makeup of ZIKV may be causing this change.

Since the first discovery of ZIKV in monkeys in Uganda, in 1949, infections in animals and humans have been incidentally recorded from Africa ever since. The virus was imported by unknown route to Asia, where it was first detected in Malaysia in 1969. Few infections have been recorded for the period of 1998 and 2007. In the early 1980s, serological evidence suggested the virus had spread in Asia to at least Malaysia, Indonesia, India, the Philippines, Thailand, and Vietnam (reviewed in [6]). Clinical cases from those countries from that period were mild, and outbreaks remained limited in size. A large outbreak in 2007 in Yap Island suggested that the virus could spread more rapidly in these island populations. An even larger outbreak in French Polynesia, during 2013–2014, reported at least two cases with severe clinical symptoms: the first case of GBS as well as transmission of the virus from a pregnant patient to her baby [7, 8]. More details describing an increase in virulence observed with ZIKV infections over time have been previously reviewed [6, 9]. As the authors of the latter review stated, there were fewer than 20 reported cases of ZIKV infection between 1947 and 2006, but already 333 confirmed cases in the Yap Island outbreak – since then, numbers have exploded as ZIKV reached Brazil and spread from that country to develop into the current pandemic. The first reports of infection-related birth defects came from Brazil, and this severe complication has since been reported from other countries as well, including the US [10], often with direct epidemiological links to Brazil, such as travel-associated cases, or sexual intercourse with a traveller.

Not only the clinical manifestations of the virus have changed, its mode of transmission also seems to be changing, as cases caused by sexual transmission are increasing [11], and the first cases of human-to-human transmission have now been described [12, 13]. It appears that during the past decade the infectivity of ZIKV increased, resulting in larger outbreaks, and symptoms got more severe, but no evidence of microcephaly has been observed until the virus hit Brazil. The geographic spread, with necessary adaptation to novel host reservoirs, together with novel transmission routes, has imposed severe and multiple bottlenecks on the viral population. Together with the high mutation rate that is typical for RNA viruses, it can be assumed that at least some of the emerging novel characteristics of ZIKV have a genetic basis, driven by evolutionary selective pressures. Indeed, multiple publications [9, 14, 15] have demonstrated that ZIKV now comprises of three sub-lineages: the original African lineage, the Asian lineage to which the mosquito isolates originating in 1966 in Malaysia, and human isolates from Micronesia, Philippines, Cambodia, Thailand and Singapore belong, and the Brazilian lineage, which includes isolates from French Polynesia (all isolated between 2007 and 2014) and all recent isolates (some publications combine the latter two lineages and describe these collectively as ‘Asian’).

We searched for published evidence that the virulence of ZIKV has changed during the recent past. There are relatively few studies that have compared recent Brazil-type strains with historical isolates. The latter, when still available, may have undergone multiple passaged through tissue-culture cells or through mice, possibly resulting in an adapted or crippled virus. An in vitro model using brain organoids was used to demonstrate that two ZIKV isolates, from Guatemala and French Polynesia, were able to infect human brain cells [16]. Although the work was not designed to investigate if there has been a recent increase in ZIKV virulence compared with the original African lineage, differences were observed between the two strains. The question was further addressed using in vitro infection of human astrocytes in which a strain of the African lineage was compared to an isolate from French Polynesia (described as representing the ‘Asian lineage’) [17]. Differences were detected between the two strains, with the African isolate resulting in 100 times more viral RNA due to a slower astrocyte’s anti-viral response, though both strains produced equal amounts of virus titers [17]. These results suggest the astrocytes were less well equipped to remove the African strain than the Brazilian strain.

An important study compared three ZIKV isolates: one from Mexico, which we describe here as part of the Brazilian lineage though the authors describe it as ‘Asian’, one from Cambodia and an isolate belonging to the African lineage [18]. These virus strains were allowed to infect a cell line derived from human fetal brain-derived neural stem cells. All three ZIKV isolates infected the stem cells equally and resulted in reduced cell proliferation. However, only the isolate from Mexico decreased neuronal differentiation, which can be taken as an important step in the development of a fetal brain [18]. We acknowledge this as important supportive evidence, in addition to the epidemiological observations, that something in ZIKV has changed since it reached Brazil.

Several research articles have presented possible explanations for the change in virulence that occurred since the virus left Africa, and for the teratogenic effects of ZIKV once it reached South-America. One of the host factors that received a lot of interest in this respect is AXL, which was proposed as the cellular receptor for ZIKV [19, 20]. However, since it was demonstrated that AXL inactivation does affect viral uptake in cerebral organoids [21], we did not pursue this direction any further.

The aim of this work was to find genetic evidence of changes in ZIKV that could explain its increased virulence. Our approach was to assess published data suggesting ZIKV lineages differ in virulence, in support to the epidemiological evidence, could be validated by bioinformatics analyses using the largest ZIKV genomes dataset analyzed to date.

## Results and discussion

### Mechanistic explanations dependent on immunological characteristics

Several mechanistic explanations for the increased virulence of ZIKV infections from Brazil compared to historical cases depend on a role of the immune system, more specifically on the presence of linear or discontinuous epitopes (recognized by antibodies or acting via cellular immunity) that must be conserved in the Asian or Brazilian types of ZIKV but differ from the historical strains from Africa. Such changes in epitopes should be consistently present in amino acid sequence comparisons.

**Fig. 1:**
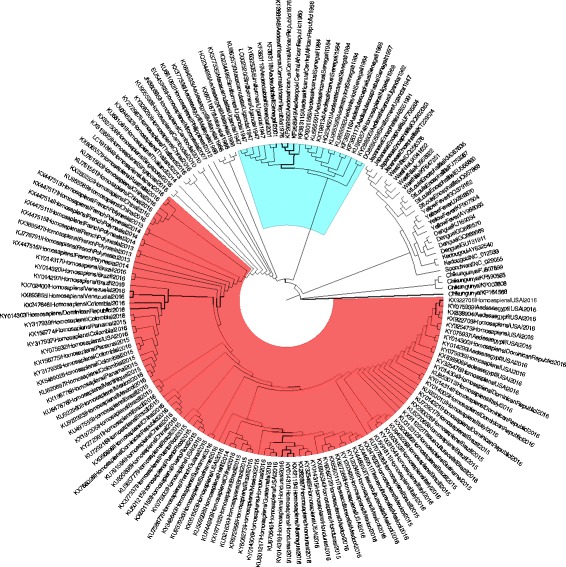
Comparison of Zika, Spondweni, Dengue, Japanese encephalitis, Kedougou, St. Louis encephalitis, West Nile, Yellow fever, and Chikungunya proteomes. Several complete proteomes were included for each species in the family *Flaviviridae* and for the species Chikungunya except for Zika virus for which all proteomes available were included. The sublineages are colored with African (cyan) and Brazilian (red). The tree is an unscaled maximum likelihood tree of complete proteomes

The 138 ZIKV complete proteome sequences that were publicly available at the time of analysis were compared by a phylogenetic tree in Fig. 1. The proteomes of several members of other species within the *Flaviviridae* family as well as Chikungunya were added for comparison. In Fig. 1, all ZIKV proteomes formed a distinct cluster, even though the virus immunologically cross-reacts with antibodies against Dengue [22]. Immunological cross-reactivity between ZIKV and DENV has been discussed in the literature with two opposing effects. T-cell memory resulting from pre-exposure to DENV might (partially) inactivate ZIKV, thus helping the immune system to limit the infection [23]. It has been shown that antibodies to envelope protein E are less specific for ZIKV and more likely cross-react to DENV or other virus species than antibodies against proteins NS1 or NS5 [24, 25]. However, this immunological cross-reactivity may actually worsen the infection, via a process named antibody-dependent enhancement [22]. Although the human population in Brazil might have been pre-exposed to DENV prior to arrival of ZIKV, the same would have been true for people in Asia and Africa (where microcephaly was not observed), while populations in the US, not frequently pre-exposed to DENV, nevertheless suffer from an increased risk of birth defects as a result of ZIKV infection. Thus, even if antibody-dependent enhancement plays a role in ZIKV infection, it does not explain the observed teratogenic effects [10].

Cellular immune responses that are important to combat viral infections require the activity of CD8^+^ T-cells. Cross-reactivity of these cells to ZIKV and DENV epitopes was demonstrated in mouse experiments [26]. During pregnancy, the CD8^+^ T-cell response is weakened, at least in mice, which may enhance the chance the virus reaches the fetuses [27]. It is possible that this also occurs in humans.

Epitopes for MHC class I peptides have been predicted in silico [28, 29]. We checked if the four predicted epitopes identified in [28] (all in protein E) are conserved. Epitope YRIMLSVHG is nearly completely conserved in all ZIKV genomes (only one mismatch in a Senegal 2001 isolate (KF383118)), but it is positioned close to a glycosylation site, which may not be favorable. Epitope VLIFLSTAV, located at the C-terminal end of protein E, is specific for the Brazilian/Asian isolates. The other two epitopes (MMLELDPPF and GLDFSDLYY) are conserved in all ZIKV genomes. Only the latter was detected by the more extensive in silico epitope prediction [29], which resulted in 49 predicted B-cell epitopes, of which 21 were located in protein E, 6 in NS3 and 22 in NS5. Compared to [28], two epitopes (partly) overlapped: Y*RIMLSVHG*SQ and GLD*FSDLYY*LTM (overlap in italics). Mirza et al. also scored proteins for locations with high surface accessibility, surface flexibility and hydrophilicity (all by means of amino acid sequence predictions), but these findings were not related to the predicted epitopes. Thirty epitopes were predicted for T-cells (10 in protein E, 5 in NS3 and 15 in NS5) [29]. Discontinuous epitopes were also predicted but this just resulted in a long list of single amino acids in proteins E, NS3 and NS5, which isn’t very helpful for vaccine development. Three of the predicted T-cell epitopes in protein E were proposed to have strong binding capacity: MAEVRSYCY, FSDLYYLTM, and TMNNKHWLV. We checked how strongly conserved these are; the first is not conserved in isolates from Guatemala and for the last there are mismatches in at least two genomes, but the middle one is 100% conserved.

### Did ZIKV undergo recombination resulting in increased virulence?

Since ZIKV shares the mosquito host with a number of other flavivirus species, it is in principle possible that recombinations have taken place between the viral RNA genomes of different species, although Musso and Gubler considered this an unlikely scenario [9]. Recombinations have been proposed by at least two research groups to explain the increase in ZIKV virulence [30, 31]. Faye et al. concluded from a comparison of 43 ZIKV genomes including the African lineage and isolates from Malaysia and Micronesia, that the virus had undergone several recombinations during its stay in Senegal and Côte d’Ivoire [30]. Han et al. concluded, after comparing 32 genomes, that recombination may have taken place in Brazilian strains, as some parts of their genome resembled isolates from Suriname and others French Polynesian isolates [31].

We used the DNA sequences of the 196 ZIKV genomes to analyze for evidence of recombination. This confirmed the findings by Faye et al. [30], that five genomes belonging to the African lineage were potential recombinants, with parental strains also from that lineage, as summarized in Additional file 1: Table S1. However, we could not detect recombination events within or between Asian and Brazilian lineages. From this we conclude that recombination events have not resulted in genetic changes that increased the virulence of ZIKV.

### Did positive selection result in genetic variants with increased virulence?

Several publications have produced phylogenetic trees that clearly separated the African from the Asian lineage, and further placed the Brazilian lineage as offspring of the Asian lineage [9, 14, 15, 32]. These observations were used to postulate that particular genetic variants might have been under positive selection, and thus be enriched in viral populations [14, 15, 32]. Based on analysis of 33 ZIKV genomes, it was questioned if the Brazilian lineage was truly derived from the Asian lineage [33]. Such conclusions must be weighed against the natural variation occurring in the viral population, and observations become more accurate with larger datasets.

**Fig. 2:**
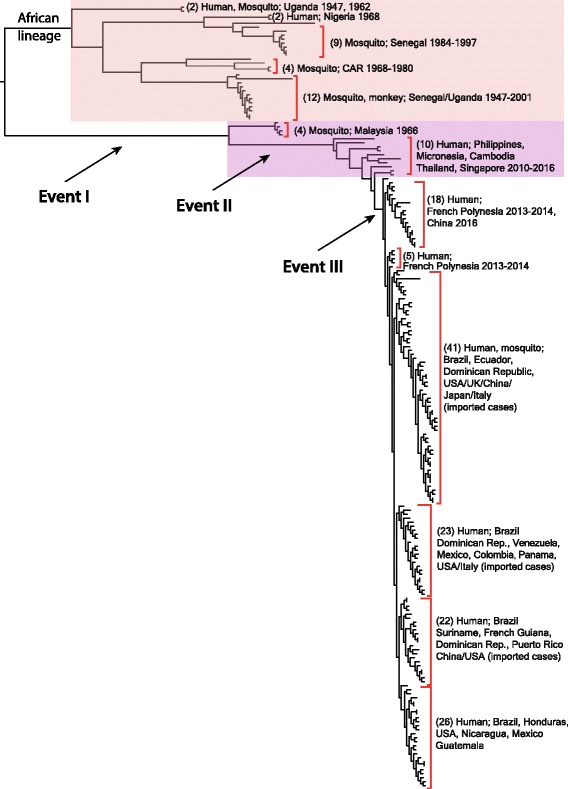
Maximum likelihood tree of 196 ZIKV complete coding DNA sequences. The tree was rooted by root-to-tip regression analysis, meaning that the branch length is most correlated with isolation date under the assumption of a strict molecular clock (correlation = 0.95). Three evolutionary events indicated with Event I, II, and III (with 100% bootstrap support, data not shown) were examined for adaptive evolution. The African and Asian lineages are color shaded. A version of the tree with branch labels is presented as Additional file 2: Figure S1

We produced a phylogenetic tree of 196 ZIKV complete coding sequences (Fig. 2). The tree identified three main events: Event I separates all African Zika genomes from the rest. Event II splits off four 1966 Malaysian isolates and all African Zika genomes from the rest. Event III separates a large cluster containing all French Polynesia isolates, all Brazil 2015–2016 isolates plus all other recent isolates from countries to where the virus has spread since.

Non-synonymous mutations were analyzed to determine whether such changes were the result of adaptive evolution, using the branch-site model implemented in PAML [34] that has been used previously [15]. Our analysis focused on the three lineages noted by Events I, II, and III in Fig. 2, and the results are presented in Table 1. For these key Events, not a single gene was subject to statistically significant positive selection. In our larger dataset, the three amino acid positions in proteins NS4B and the two in NS5 that were previously reported to be under positive selection [15] were not statistically significant.

**Table 1:** Adaptive evolution analysis.

Protein	Reference protein		Residue in the Event I, II, and III				Probability (ω > 1)
	Accession	Site	Before Event I	After Event I	After Event II	After Event III	
C	YP_009227206	R101	R	K	K	K	0.508
		I110	I	V	V	V	0.519
NS2A	YP_009227200	V208	I(12), V(3)	L	L	L	0.578
pre-M	YP_009227197	V31	V	V	M	M	0.572
NS2A	YP_009227200	A143	A	A	V	V	0.621
NS4B	YP_009227204	L49	L	L	F	F	0.603
		V184	V	V	I	I	0.629
		L186	L	L	S	S	0.841
NS5	YP_009227205	S703	S	S	D	D	0.941
pre-M	YP_009227197	S17	S	S	S	N	0.696

The branch-site model was performed with the pre-specified lineages noted by Events I, II, and III in Fig. 2. The positions are inferred from the proteins of reference genome strain MR 766 (NC_012532), for which the accession numbers are specified. For the site V208 in NS2A, two amino acid letters were observed before Event I, and numbers in parenthesis indicate their occurrence

The table reports all sites under positive selection whose posterior probability of ω >1 is at least over 0·5 where ω is the ratio of non-synonymous rate to synonymous rate. The A148P mutation in pre-M previously noticed [32] was not found under positive selection in our analysis, and although those authors noticed relative high variability in pre-M, the substitution we identified was not scored in their analysis of 41 genomes [32]. Mutation analysis of ZIKV is most interesting for mutations that would induce changes in N-linked glycosylation sites in envelope protein E, but these were not found. Our results contrast to previously reported findings that position 154 of protein E was N-glycosylated in the Brazilian lineage [9]. We observed that the correlation is not that strong, as only five of the 25 African genomes lack this N154. In fact, our analysis did not identify any positive selection for protein E. This is unexpected, since this surface protein is considered to be under immune-selection during infection. However, we identified ten amino acid residues that were indicative of adaptive evolution: two in the protein C, two in pre-protein M, and six in non-structural proteins by model M8 (described in the Method section). In accordance with an analysis based on 46 genomes [35], our data report far fewer mutations under positive selection in the Brazilian lineage than have previously been reported [14]. Our findings are more conservative because they only report substitutions beyond the probability threshold of the applied model.

A publication in 2017 described a T233A mutation in protein NS1 in an isolate from a neuropathy case [36]. However, this mutation is not conserved in the Brazilian lineage and is found only once in our dataset. It is unlikely to have been responsible for all neuropathy cases described so far.

We further analyzed the predictive effect of the positively selected amino acid change in NS5, the RNA-dependent RNA polymerase, by analyzing its Pfam domain. The mutation resulted in an improved match to the PfamA domain PF00972. This could result in an increase in its enzyme activity (which is only a hypothesis at this stage). If that hypothesis proves to be correct, it could potentially result in more rapid production of positive-strand RNA copies, and this would exponentially increase the number of negative strand genome replicates, which are typically produced in 10 times excess compared to the positive strand. In this context, it is interesting to note that ZIKV strains of the Brazilian lineage have been shown to replicate faster in vitro [37].

### Is RNA degradation impaired in infected cells?

A different mechanistic explanation was proposed, namely that the RNA genome of ZIKV could be unusually resistant to degradation in the infected cell [38]. If viral RNA fragments could resist exonuclease degradation, this would dysregulate RNA degradation in the cell. The authors observed that ZIKV RNA folding increased resistance to RNAse Xrn1 [38]. However, the sequence they propose to be responsible for this resistance is 100% conserved in our complete dataset (for those genomes that recorded the non-coding 3′-end of the genome), so the observation doesn’t explain why virulence of ZIKV has increased over time, or why the Brazilian lineage in particular causes microcephaly.

### Has ZIKV adapted to new hosts?

In a recent review, two possible mechanisms responsible for the recent ZIKV pandemic were discussed: “(i) evolution for enhanced urban transmission via adaptation to mosquito vectors, or for enhanced human infection to increase amplification, or (ii) the stochastic introduction of ZIKV into large, naive human populations in regions with abundant *Aedes aegypti* populations, leading to enough rare, severe infection outcomes for their first recognition.” [39]. Since viral adaptation will leave recognizable traces in the viral genome, we tried to validate the first proposed mechanism by bioinformatics analysis.

For a start, it has been suggested that Retinoic Acid Response Element (RARE) sequences present in the ZIKV genome would upset the neural development of infected fetal cells [40]. This sequence is the response element of retinoic acid, an early neural tube developmental marker. The authors determined that 17 ZIKV genomes contained between 2 and 4 copies of this RARE element, with members of the Brazilian lineage containing 4 copies. However, the authors only searched for the element in the RNA sequence of the virus [40], while the mechanistic explanation they provide would also apply to the cDNA produced in an infected cell. We therefore searched to determine the presence of RARE sequences in both strands. This changed the numbers some, as we observed 4 to 5 copies in isolates of the African lineage, 5 to 7 copies in the Asian lineages and 6 to 7 copies in the Brazilian lineage, but it confirmed the tendency of increasing RARE elements in the more virulent Brazilian lineage.

**Fig. 3:**
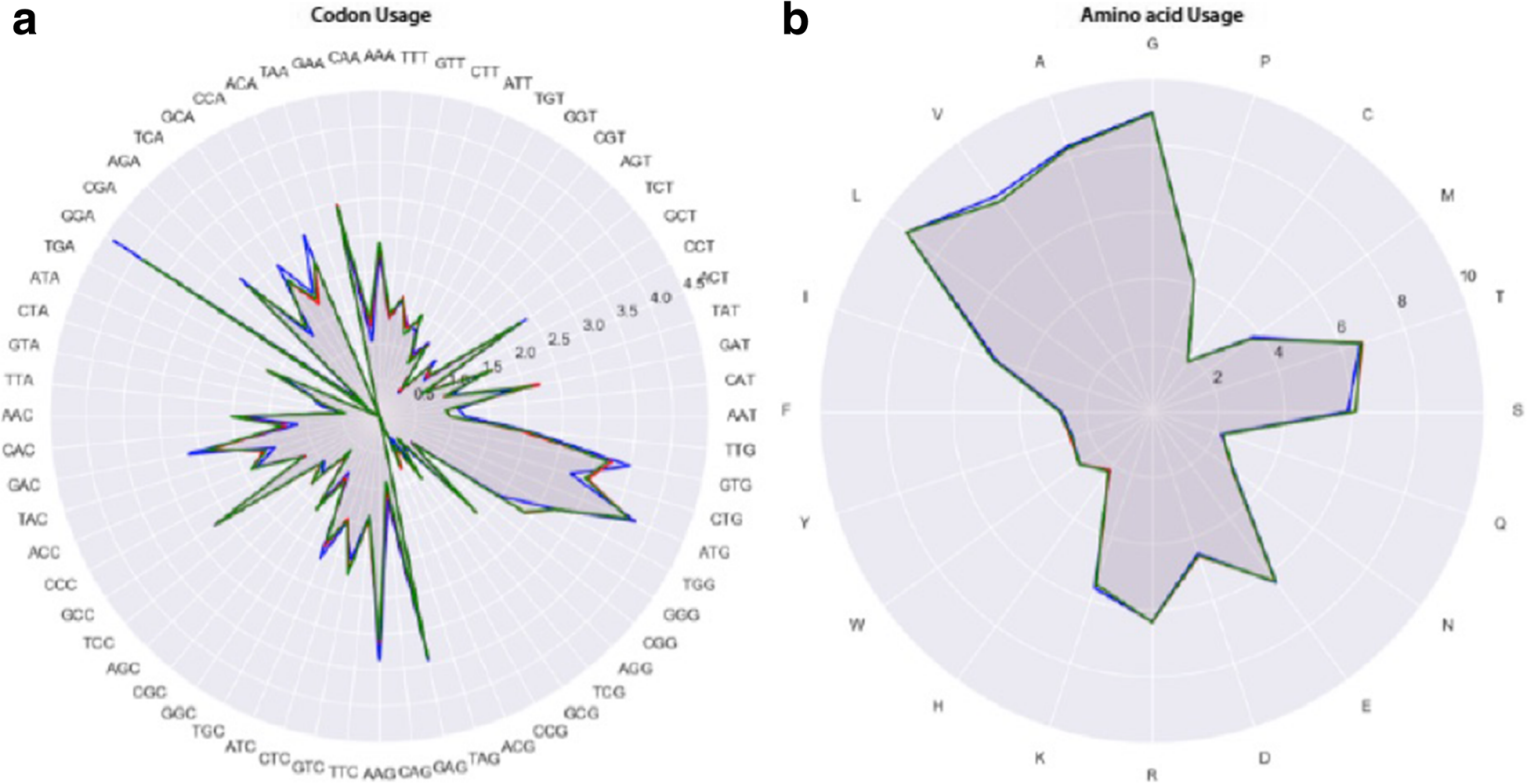
Codon usage (**a**) and amino acid usage (**b**) of the African (blue), Asian (red) and Brazilian (green) ZIKV lineages

We next investigated tetramers, since it has been observed that viruses adapt their genomes according to the host in which their main population propagates, and such adaptation can be visualized by the frequency of tetramers [41]. K-mer analysis with K values from 1 to 10 was applied to seek for patterns; the results are summarized in Additional file 3: Table S2. This revealed no difference in frequency of dimer, trimer or tetramer frequency between genomes of the African and Asian lineages, but differences between the African and Brazilians lineages, and (with the exception of dimers) between Asian and Brazilian genomes were observed. The lack of a significant difference between the African and Asian genomes was further apparent for hexamers. This is probably due to codon usage constraints, which (when in coding regions and in frame) overlap with trimer frequency. Indeed, when codon usage was compared (Fig. 3a), minor differences were identified between the three lineages, resulting at the protein level in a slight though significant increase in the usage of serine (Fig. 3b) (*p*-value of 6e-13), and a small decrease in valine (*p*-value of 4e-13).

Adaptation of codon usage in the ZIKV lineages has been described by others [42, 43]. However those analyses included fewer genomes (31 and, 46 respectively). Wang and colleagues [43] concluded that codon usage within ZIKV was shaped to fit the human host more than the mosquito vector (other hosts were not considered). Since the larger dataset analysed here identifies only minor differences between the various lineages (Fig. 3a), such adaptation cannot be responsible for the observed increase in virulence.

Tetramers are the shortest oligomers that are not strongly affected by codon usage preferences. The ratio of observed over expected tetramer frequency of all ZIKV genomes was compared to seek for evidence of genome adaptation over time. For this, expected frequencies were derived using the second order Markov model described in [44]. The results (shown in Additional file 4: Figure S2) show that that the tetramer frequency varied considerably between, but not within groups representing the different historical and geographical clusters of the phylogenetic tree. This is most likely due to viral adaptation to different sylvatic or vector hosts, which may vary between countries and continents. However, the French Polynesia/China cluster together with the Brazilian cluster report more or less constant tetramer frequencies, indicating that since then, the main viral population has adapted to propagation in a limited and constant range of hosts.

**Fig. 4:**
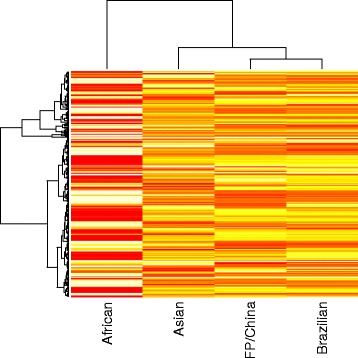
Heatmap showing the relationship of the African, Asian and Brazilian lineages based on the average observed frequencies of tetramers. In this analysis, isolates from French Polynesia and China, which all clustered together in Fig. 2, are analysed separately, labelled with FP/China

Figure 4 shows a heatmap of the tetramer frequency, visualizing that genomes of to the Brazilian lineage are quite different to African strains, and somewhat closer to the Asian cluster, in agreement with their phylogenetic relationship. For this analysis, isolates from French Polynesia and China that clustered closely in Fig. 2 were separately analysed. The similarity in tetramer frequency between this group and the Brazilian lineage is striking, and indicates that the adaptation process occurred in French Polynesia. This dating fits with an estimate that the Brazilian lineage arose between 2011 and 2013 [14, 35], and suggests that the change in tetramer frequencies must have occurred rapidly.

**Fig. 5:**
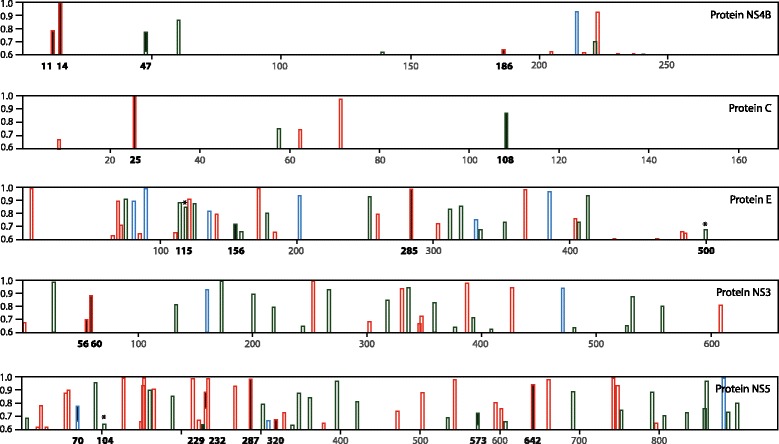
Predicted phosphorylation scores above the threshold of 0.6 for amino acid residues in Zika proteins. For each protein, phosphorylation scores above the threshold of 0.6 for amino acid residues Serine (red), Threonine (green) and Tyrosine (blue) are shown. Changes in scores between the African and Brazilian lineages are shown as black filled columns (additional or higher-score phosphorylation sites present in Brazilian lineage) and asterisks (sites which resulted in a decreased phosphorylation score in Brazilian lineage compared to African lineage proteins)

The differences between African, Asian, and Brazilian lineages were further compared based on Zika protein property predictions, to evaluate their impact on protein function. No noticeable changes between African, Asian, and Brazilian lineages were observed in predicted protease cleavage, glycosylation sites, signal peptides or transmembrane domains. The positively selected mutations in NS4B (summarized in Table 1) only marginally influenced scores for Pfam domain PF01349 that may impact its function. Within the African and Brazilian lineages, phosphorylation sites of individual proteomes were strongly conserved (data not shown). However, remarkable changes were noted in phosphorylation sites between these lineages in proteins NS4B, C, E, NS3 and NS5 in Fig. 5. For example, the African lineage has a total of 11 conserved amino acid residues in NS4B reaching phosphorylation site scores above the threshold (5 Serine, 5 Threonine, and 1 Tyrosine, of which 3 resulted in high scores of >0.8), while the Brazilian lineage had 14 conserved sites where the three of them are novel Serine (S) phosphorylation sites and one of novel sites was also identified by the positive selection analysis (L186S, Table 1). Another (N11S) produced an additional putative phosphorylation site in the N-terminus of NS4B, while Leucine to Phenylalanine change at position 49 resulted in a higher score for neighboring Threonine (T47). These observations are relevant in view of the in vitro observation that Zika protein NS4B (together with NS4A) induces autophagy in fetal neural stem cells, due to inhibition of Akt-mTOR signaling [45]. The phosphorylation site analysis of all ZIKV proteins combined identified loss of 2 phosphorylation sites but 14 sites were novel phosphorylation sites in the Brazilian lineage (summarized in Table 2). As a result, the proteins of ZIKV isolates belonging to the Brazilian lineage are likely to be stronger phosphorylated than the corresponding proteins of the African lineage. The analysis was refined by comparing Brazilian strains with the four Malaysian 1966 strains (Asian1) and the other Asian strains (Asian2). The Malaysian strains differed in 6 positions, having 1 extra and 5 fewer phosphorylation sites compared to the Brazilian strains (Table 2). However, there were no differences between Brazilian and Asian strains excluding the Malaysia isolates in terms of novel phosphorylation sites (Table 2). Of note is the increase in phosphorylation sites in NS5, which is not only the largest protein of ZIKV but also underwent the most extensive changes in phosphorylation sites: 1 site as lost and 4 were added as the African lineage evolved in the Brazilian lineage, with the Asian1 strains in between. Whether these changes in phosphorylation sites affected the activity of this enzyme remains to be assessed.

**Table 2:** Novel phosphorylation sites

	C	M	E	NS1	NS2A	NS2B	NS3	NS4A	NS4B	NS5
African-Brazilian	0:2	0:0	1:3	0:0	0:0	0:0	0:2	0:0	0:3	1:4
Asian1-Brazilian	0:0	0:0	0:1	0:0	0:0	0:0	0:0	0:0	0:2	1:2
Asian2-Brazilian	0:0	0:0	0:0	0:0	0:0	0:0	0:0	0:0	0:0	0:0

The phosphorylation sites with scores >0.6 were only considered. The Asian1 group consists of four Malaysia 1966 isolates, and the Asian2 consists of the rest of isolates from the Asian lineage

That amino acid changes have added phosphorylation sites or increased the likelihood of phosphorylation the proteins of Brazilian strains is an important finding, and we believe this could have significant effects in neural cells. The demonstration in vitro of neurogenesis inhibition and induced autophagy by Zika infection on isolated fetal neural stem cells would provide a likely mechanism for neuropathy [45]. In particular, proteins NS4A and NS4B were reported to be responsible for inhibition of Akt-mTOR signaling, which is essential for neurogenesis, by reducing Akt phosphorylation. Moreover, NS4A/NS4B induced autophagy, which promotes viral replication, by reducing mTOR phosphorylation. Thus, we hypothesize that increased phosphorylation of NS4B and possibly other ZIKV proteins in the Brazilian lineage contributed to the pathophysiology in neural tissue.

## Conclusions

After evaluating a number of proposed mechanistic explanations for the increased virulence and recent teratogenic and neuropathological effects of ZIKV, a number of these can be rejected, based on non-consistent observations in the largest ZIKV genome set analyzed to date. A number of observations remain valid that, possibly in combination, might be responsible for the observed disease characteristics of what once seemed to be a mild infection. Notably, the increase in RARE sequences present in the ZIKV genomes of the Brazilian lineage, their tetramer adaptation to fit a narrower host range of mosquitoes and humans, and positively selected mutations in protein NS5 may have resulted in a viral population that is better equipped to replicate in the human host. In addition, mutations in NS4B may result in higher phosphorylation status of viral proteins, upsetting Akt-mTOR signaling in infected fetal neural cells. The combination of these features may be at the basis of the accumulatively changed characteristics of ZIKV since it left Micronesia.

## Methods

### *Flavivirus* and *Chikungunya* complete proteomes

For comparative proteome analysis of *Flavivirus* members, all available complete proteomes were downloaded from GenBank on July 1, 2016 that resulted in 3300 Dengue fever virus (DENV) complete proteomes, 183 Japanese encephalitis, 9 St. Louis encephalitis, 1014 West Nile, 122 Yellow fever, one Kedougou and one Spondweni virus proteomes. Complete proteomes of Chikungunya virus (an Alpha virus belonging to the *Togaviridae* family) were added since this virus produces similar clinical features to ZIKV. To reduce computational costs, we randomly choose four members for each species except for ZIKV for which all proteomes (138 unique ZIKV proteomes) available were included. We parsed the protein sequences from each GenBank file and concatenated these to generate a complete proteome. We aligned complete proteomes using MUSCLE [46], and then built the maximum-likelihood tree shown in Fig. 1 using RAxML [47], automatically testing models with and without empirical base frequencies.

### Zika virus complete coding sequences

A total of 202 ZIKV complete coding sequences available from the Virus Variation database at NCBI on March 16, 2017 were downloaded. Among them, 196 ZIKV complete coding sequences were chosen based on their quality defined by lack of bases not being A, T, G, and C. As metadata we recorded the isolation year, not the date when isolates had been sequenced, and, in case of travel-associated cases, the country of presumed infection. We aligned complete coding sequences using MUSCLE [46], and then built the maximum-likelihood tree using FastTree [48] shown in Fig. 2, where the tree was rooted based on a root-to-tip regression analysis [49] with dated tips that branch length from the root is most compatible with the assumption of a strict molecular clock.

### Recombination analysis

The recombination detection program RDP4 [50] was used with default settings (window size: 30 bp). Recombination events, which refer to the formation of chimeric sequences from parent genomes, were inferred by seven independent methods: RDP, GENECONV, BootScan, MaxChi, Chimaera, SiScan, and 3Seq, all implemented in RDP4.

### Adaptive evolution analysis

Positive selection analysis was performed with the branch-site model, using the application codeml implemented in PAML [34]. First, ten mature peptides were inferred from multiple sequence alignment of Zika complete coding sequences based on the annotation of the reference genome (NC_012532). For each protein gene, we then generated a non-redundant dataset of coding sequences creating a non-redundant dataset of 46 sequences for gene C, 66 for M, 117 for E, 94 for NS1, 88 for NS2A, 53 for NS2B, 115 for NS3, 49 for NS4A, 23 for 2 K, 85 for NS4B, and 147 for NS5. A multiple alignment of nucleotide sequences was produced, guided by amino acid information using TranslatorX [51] for each gene. Next, with the non-redundant dataset of each gene, a phylogenetic tree was constructed based on amino acid guided nucleotide sequence alignment using PhyML [52] with the best-fit model identified by jModelTest [53]. For sites under positive selection in the specified lineages (noted by Event I, II, and III on the tree in Fig. 2), we employed null and alternative models defined in the branch-site model A implemented in codeml [34]. We compared the alternative model against the null model by means of a likelihood-ratio test (LRT) and calculated the *p*-value under Chi-square distribution for each gene. Not a single gene was identified with a p-value of LRT statistic <0.05. However, we recorded those amino acid positions with ω >1 whose posterior probability, as calculated by the Bayes Empirical Bayes (BEB) method, is at least over 0.5. However, trees of genes M and C resulted in a different tree topology compared to the tree in Fig. 2: for example, Malaysia isolates were positioned inside the Brazilian lineage. In such a case (Event III for gene C, Event II and III for gene M), we alternatively performed positive selection analysis with a tree generated with an alignment of complete coding sequences for a non-redundant dataset. Also, the multiple sequence alignment of gene NS5 (147 NS5 sequences and 2709 sites) was too large to run codeml in a timely manner on the available hardware so that we used an alternative dataset of 82 NS5 sequences.

### Protein properties analysis

Functional domain(s) were identified through the PFAM databases [54] using gathering cut-off. Trans-membrane region(s) were identified by TMHMM v2.0 [55]. For signal peptide identification SignalP v4.1 [56] was used. Glycosylation site(s) were identified by NetNGlyc v1.0 [57] and by NetOGlyc v4.0 [58]. Phosphorylation scores were calculated by NetPhos v3.1 [59]. Cleavage sites of the polyprotein were reported according to [60].

## Additional files

Additional file 1: Table S1. Recombination analysis. The recombination events detected by RDP4 with default setting are listed, ordered for decreased consensus level of results obtained with seven different methods: R (RDP), G (GENECONV), B (BootScan), M (MaxChi), C (Chimaera), S (SiScan), T (3Seq). ‘+’ means detected, and ‘-’ not detected by the method. (DOCX 32 kb)

Additional file 2: Figure S1. Maximum likelihood tree of 196 ZIKV complete coding sequences. The tree was rooted by root-to-tip regression analysis, meaning that the location is most compatible with the assumption of a strict molecular clock (correlation = 0.95). The branches are labeled with accession number, host species (African and Asian lineage only), country and collection year of isolation. All isolates in the Brazil lineage were from humans with the exception of 14 USA isolates (KY075938, KY014324, KX838904, KY075939, KY014323, KX838905, KY075937, KY014322, KX838906, KX922708, KY014322, KX838906, KX922708, KY014299) and two Mexican isolates (KX446950, KX446951), which were isolated from *Aedes* mosquitoes. (DOCX 58 kb)

Additional file 3: Table S2. K-mer analysis for the three ZIKV lineages. The *p*-values, calculated by one-sample Wilcoxon test, are given for pairs of lineages. (DOCX 30 kb)

Additional file 4: Figure S2. Ratio of observed over expected tetranucleotide frequency in ZIKV genomes in historical isolates up to 2014 (top) and from the French Polynesian outbreak and onwards (bottom). The genomes are ordered according to their position in the phylogenetic tree, with clusters separated by dotted lines. A few individual genomes are listed below the panels for reference. The last two genomes belonging to the Asian lineage 2010–2014 shown in the top panel (far right) are repeated in the lower panel (far left). The tetranucleotide with the highest and lowest frequency in the Brazil lineage are shown by bold orange (TAAT) and red (TATC) lines, respectively. (DOCX 378 kb)

## References

[1] Verma R, Sahu R, Holla V (2014). Neurological manifestations of dengue infection: a review. J Neurol Sci.

[2] Vellozzi C, Iqbal S, Broder K (2014). Guillain-Barre syndrome, influenza, and influenza vaccination: the epidemiologic evidence. Clin Infect Dis.

[3] Melo AS, Aguiar RS, Amorim MM, Arruda MB, Melo FO, Ribeiro ST (2016). Congenital Zika virus infection: beyond neonatal Microcephaly. JAMA Neurol.

[4] Nayak S, Lei J, Pekosz A, Klein S, Burd I (2016). Pathogenesis and molecular mechanisms of Zika virus. Semin Reprod Med.

[5] Fagbami AH (1979). Zika virus infections in Nigeria: virological and seroepidemiological investigations in Oyo state. J Hyg (Lond).

[6] Armstrong N, Hou W, Tang Q (2017). Biological and historical overview of Zika virus. World J Virol.

[7] Oehler E, Watrin L, Larre P, Leparc-Goffart I, Lastere S, Valour F, et al. Zika virus infection complicated by Guillain-Barre syndrome--case report, French Polynesia, December 2013. Euro Surveill. 2014;19(9). 10.2807/1560-7917.es2014.19.9.20720 24626205

[8] Besnard M, Lastere S, Teissier A, Cao-Lormeau V, Musso D. Evidence of perinatal transmission of Zika virus, French Polynesia, December 2013 and February 2014. Euro Surveill. 2014;19(13). 24721538

[9] Musso D, Gubler DJ (2016). Zika virus. Clin Microbiol Rev.

[10] Cragan JD, Mai CT, Petersen EE, Liberman RF, Forestieri NE, Stevens AC (2017). Baseline prevalence of birth defects associated with congenital Zika virus infection - Massachusetts, North Carolina, and Atlanta, Georgia, 2013-2014. MMWR Morb Mortal Wkly Rep.

[11] Basu R, Tumban E (2016). Zika virus on a spreading spree: what we now know that was unknown in the 1950’s. Virol J.

[12] Swaminathan S, Schlaberg R, Lewis J, Hanson KE, Couturier MR (2016). Fatal Zika virus infection with secondary nonsexual transmission. N Engl J Med.

[13] Yin Y, Xu Y, Su L, Zhu X, Chen M, Zhu W (2016). Epidemiologic investigation of a family cluster of imported ZIKV cases in Guangdong, China: probable human-to-human transmission. Emerg Microbes Infect.

[14] Pettersson JH, Eldholm V, Seligman SJ, Lundkvist A, Falconar AK, Gaunt MW (2016). How did Zika virus emerge in the Pacific Islands and Latin America?. MBio.

[15] Sironi M, Forni D, Clerici M, Cagliani R (2016). Nonstructural proteins are preferential positive selection targets in Zika virus and related Flaviviruses. PLoS Negl Trop Dis.

[16] Gabriel E, Ramani A, Karow U, Gottardo M, Natarajan K, Gooi LM (2017). Recent Zika virus isolates induce premature differentiation of neural progenitors in human brain Organoids. Cell Stem Cell.

[17] Hamel R, Ferraris P, Wichit S, Diop F, Talignani L, Pompon J (2017). African and Asian Zika virus strains differentially induce early antiviral responses in primary human astrocytes. Infect Genet Evol.

[18] McGrath EL, Rossi SL, Gao J, Widen SG, Grant AC, Dunn TJ (2017). Differential responses of human fetal brain neural stem cells to Zika virus infection. Stem Cell Rep.

[19] Hamel R, Dejarnac O, Wichit S, Ekchariyawat P, Neyret A, Luplertlop N (2015). Biology of Zika virus infection in human skin cells. J Virol.

[20] Faizan MI, Abdullah M, Ali S, Naqvi IH, Ahmed A, Parveen S (2016). Zika virus-induced Microcephaly and its possible molecular mechanism. Intervirology.

[21] Wells MF, Salick MR, Wiskow O, Ho DJ, Worringer KA, Ihry RJ (2016). Genetic ablation of AXL does not protect human neural progenitor cells and cerebral Organoids from Zika virus infection. Cell Stem Cell.

[22] Paul LM, Carlin ER, Jenkins MM, Tan AL, Barcellona CM, Nicholson CO (2016). Dengue virus antibodies enhance Zika virus infection. Clin Transl Immunol.

[23] Rivino L, Lim MQ (2017). CD4^+^ and CD8^+^ T-cell immunity to dengue - lessons for the study of Zika virus. Immunology.

[24] Wong SJ, Furuya A, Zou J, Xie X, Dupuis AP, Kramer LD (2017). A multiplex microsphere immunoassay for Zika virus diagnosis. EBioMedicine.

[25] Keasey SL, Pugh CL, Jensen SM, Smith JL, Hontz RD, Durbin AP, et al. Antibody responses to Zika virus infections in flavivirus-endemic environments. Clin Vaccine Immunol. 2017;24(4). 10.1128/CVI.00036-17 PMC5382833 28228395

[26] Wen J, Tang WW, Sheets N, Ellison J, Sette A, Kim K (2017). Identification of Zika virus epitopes reveals immunodominant and protective roles for dengue virus cross-reactive CD8+ T cells. Nat Microbiol.

[27] Winkler CW, Myers LM, Woods TA, Messer RJ, Carmody AB, McNally KL (2017). Adaptive immune responses to Zika virus are important for controlling virus infection and preventing infection in brain and testes. J Immunol.

[28] Alam A, Ali S, Ahamad S, Malik MZ, Ishrat R (2016). From ZikV genome to vaccine: in silico approach for the epitope-based peptide vaccine against Zika virus envelope glycoprotein. Immunology.

[29] Mirza UM, Rafique S, Ali A, Munir M, Ikram N, Manan A (2016). Towards peptide vaccines against Zika virus: Immunoinformatics combined with molecular dynamics simulations to predict antigenic epitopes of Zika viral proteins. Sci Rep.

[30] Faye O, Freire CC, Iamarino A, Faye O, de Oliveira JV, Diallo M (2014). Molecular evolution of Zika virus during its emergence in the 20(th) century. PLoS Negl Trop Dis.

[31] Han JF, Jiang T, Ye Q, Li XF, Liu ZY, Qin CF (2016). Homologous recombination of Zika viruses in the Americas. J Inf Secur.

[32] Wang L, Valderramos SG, Wu A, Ouyang S, Li C, Brazil P (2016). From Mosquitos to humans: genetic evolution of Zika virus. Cell Host Microbe.

[33] Kochakarn T, Kotanan N, Kümpornsin K, Loesbanluechai D, Thammasatta M, Auewarakul P (2016). Comparative genome analysis between southeast Asian and south American Zika viruses. Asian Pac J Trop Med.

[34] Yang Z (1997). PAML: a program package for phylogenetic analysis by maximum likelihood. Comput Appl Biosci.

[35] Ramaiah A, Dai L, Contreras D, Sinha S, Sun R, Arumugaswami V (2017). Comparative analysis of protein evolution in the genome of pre-epidemic and epidemic Zika virus. Infect Genet Evol.

[36] Wang D, Chen C, Liu S, Zhou H, Yang K, Zhao Q (2017). A mutation identified in neonatal Microcephaly destabilizes Zika virus NS1 assembly in vitro. Sci Rep.

[37] Liu S, DeLalio LJ, Isakson BE, Wang TT (2016). AXL-mediated productive infection of human endothelial cells by Zika virus. Circ Res.

[38] Akiyama BM, Laurence HM, Massey AR, Costantino DA, Xie X, Yang Y (2016). Zika virus produces noncoding RNAs using a multi-pseudoknot structure that confounds a cellular exonuclease. Science.

[39] Weaver SC. Emergence of epidemic Zika virus transmission and congenital Zika syndrome: are recently evolved traits to blame? MBio. 2017;8(1). 10.1128/mBio.02063-16 PMC5225313 28074023

[40] Kumar A, Singh HN, Pareek V, Raza K, Dantham S, Kumar P (2016). A possible mechanism of Zika virus associated Microcephaly: imperative role of retinoic acid response element (RARE) consensus sequence repeats in the viral genome. Front Hum Neurosci.

[41] Pride DT, Wassenaar TM, Ghose C, Blaser MJ (2006). Evidence of host-virus co-evolution in tetranucleotide usage patterns of bacteriophages and eukaryotic viruses. BMC Genomics.

[42] Butt AM, Nasrullah I, Qamar R, Tong Y (2016). Evolution of codon usage in Zika virus genomes is host and vector specific. Emerg Microbes Infect.

[43] Wang H, Liu S, Zhang B, Wei W (2016). Analysis of synonymous codon usage bias of Zika virus and its adaption to the hosts. PLoS One.

[44] Jun SR, Sims GE, Wu GA, Kim SH (2010). Whole-proteome phylogeny of prokaryotes by feature frequency profiles: an alignment-free method with optimal feature resolution. Proc Natl Acad Sci U S A.

[45] Liang Q, Luo Z, Zeng J, Chen W, Foo SS, Lee SA (2016). Zika virus NS4A and NS4B proteins deregulate Akt-mTOR signaling in human fetal neural stem cells to inhibit Neurogenesis and induce Autophagy. Cell Stem Cell.

[46] Edgar RC (2004). MUSCLE: multiple sequence alignment with high accuracy and high throughput. Nucleic Acid Res.

[47] Stamatakis A (2014). RAxML version 8: a tool for phylogenetic analysis and post-analysis of large phylogenies. Bioinformatics.

[48] Price MN, Dehal PS, Arkin AP (2010). FastTree 2 - approximately maximum-likelihood trees for large alignments. PLoS One.

[49] Drummond A, Pybus OG, Rambaut A (2003). Inference of viral evolutionary rates from molecular sequences. Adv Parasitol.

[50] Martin DP, Murrell B, Golden M, Khoosal A, Muhire B (2015). RDP4: detection and analysis of recombination patterns in virus genomes. Virus Evol.

[51] Abascal F, Zardoya R, Telford MJ (2010). TranslatorX: multiple alignment of nucleotide sequences guided by amino acid translations. Nucleic Acids Res.

[52] Guindon S, Dufayard JF, Lefort V, Anisimova M, Hordijk W, Gascuel O (2010). New algorithm and methods to estimate maximum-likelihood phylogenies: assessing the performance of PhyML 3.0. Syst Biol.

[53] Darriba D, Taboada GL, Doallo R, Posada D (2012). jModelTest 2: more models, new heuristics and parallel computing. Nat Methods.

[54] Finn RD, Coggill P, Eberhardt RY, Eddy SR, Mistry J, Mitchell AL (2016). The Pfam protein families database: towards a more sustainable future. Nucleic Acids Res.

[55] Krogh A, Larsson B, von Heijne G, Sonnhammer EL (2001). Predicting transmembrane protein topology with a hidden Markov model: application to complete genomes. J Mol Biol.

[56] Petersen TN, Brunak S, von Heijne G, Nielsen H (2011). SignalP 4.0: discriminating signal peptides from transmembrane regions. Nat Methods.

[57] Gupta R, Brunak S (2002). Prediction of glycosylation across the human proteome and the correlation to protein function. Pac Symp Biocomput..

[58] Steentoft C, Vakhrushev SY, Joshi HJ, Kong Y, Vester-Christensen MB, Schjoldager KT (2013). Precision mapping of the human O-GalNAc glycoproteome through simple cell technology. EMBO J.

[59] Blom N, Gammeltoft S, Brunak S (1999). Sequence- and structure-based prediction of eukaryotic protein phosphorylation sites. J Mol Biol.

[60] Lei J, Hansen G, Nitsche C, Klein CD, Zhang L, Hilgenfeld R (2016). Crystal structure of Zika virus NS2B-NS3 protease in complex with a boronate inhibitor. Science.

