# Supplementary material for: Suggested mechanisms for Zika virus causing microcephaly: what do the genomes tell us?
Source: BMC Bioinformatics. 2017 Dec 28;18(Suppl 14):471. doi: 10.1186/s12859-017-1894-3 (PMC5751795; doi:10.1186/s12859-017-1894-3)
Supplement: Supplementary file 1 — Recombination analysis. The recombination events detected by RDP4 with default setting are listed, ordered for decreased consensus level of results obtained with seven different methods: R (RDP), G (GENECONV), B (BootScan), M (MaxChi), C (Chimaera), S (SiScan), T (3Seq). ‘+’ means detected, and ‘-’ not detected by the method. (DOCX 32 kb) [file 12859_2017_1894_MOESM1_ESM.docx]

| **Accession nr.** | **Isolated from** | **Major parent** | **Minor parent** | **R** | **G** | **B** | **M** | **C** | **S** | **T** |
| --- | --- | --- | --- | --- | --- | --- | --- | --- | --- | --- |
| KF383117 | *Aedes*  *luteocephalus*  (Senegal, 1997) | KF383116, *A. luteochephalus* (Senegal, 1968) | KF383115, *A. africanus* (CAR, 1968) | + | + | + | + | + | + | + |
| KF383118 | *Aedes dalzieli* (Senegal, 2001) | KF383119, *A. dalzieli*  (Senegal, 2001) | KF383117, *A. luteocephalus* (Senegal, 1997) | + | + | + | + | + | + | + |
| KF383117 | *Aedes*  *luteocephalus*  (Senegal, 1997) | HQ234501, *A. africanus*  (Senegal, 1984) | KF383119, *A. dalzieli* (Senegal, 2001) | + | + | + | + | + | + | + |
| KF383117 | *Aedes*  *luteocephalus*  (Senegal,1997) | KF383116, *A. luteochephalus*  (Senegal, 1968) | KF383118, *A. dalzieli* (Senegal, 2001) | + | + | + | + | + | + | + |
| KF383116 | *Aedes*  *luteochephalus*  (Senegal, 1968) | KU955591, *A.* africanus  (Senegal, 1984) | KF383115, *A. africanus*  (CAR, 1968) | + | + | + | + | + | + | + |
| KF383118 | *Aedes dalzieli* (Senegal, 2001) | KF383119, *A. dalzieli*  (Senegal, 2001) | Unknown | + | + | + | + | + | - | + |
| KF383118 | *Aedes dalzieli* (Senegal, 2001) | LC002520, monkey, (Uganda, 1947) | Unknown | + | + | + | + | + | - | + |
| KF383118 | *Aedes dalzieli* (Senegal, 2001) | KF383115, *A. africanus*  (CAR, 1968) | Unknown | - | - | - | + | + | + | + |
| KF383116 | *Aedes*  *luteochephalus*  (Senegal, 1968) | KF383117, *Aedes luteocephalus*  (Senegal, 1997) | Unknown | + | + | + | - | - | - | - |
| KF383117 | *Aedes*  *luteocephalus*  (Senegal, 1997) | KF383116, *A. luteochephalus*  (Senegal, 1968) | LC002520, monkey, (Uganda, 1947) | - | + | + | - | - | - | - |

**Table S1.** Recombination analysis. The recombination events detected by RDP4 with default setting are listed, ordered for decreased consensus level of results obtained with seven different methods: R (RDP), G (GENECONV), B (BootScan), M (MaxChi), C (Chimaera), S (SiScan), T (3Seq). ‘+’ means detected, and ‘-’ not detected by the method.
