# Supplementary material for: Suggested mechanisms for Zika virus causing microcephaly: what do the genomes tell us?
Source: BMC Bioinformatics. 2017 Dec 28;18(Suppl 14):471. doi: 10.1186/s12859-017-1894-3 (PMC5751795; doi:10.1186/s12859-017-1894-3)
Supplement: Supplementary file 2 — Maximum likelihood tree of 196 ZIKV complete coding sequences. The tree was rooted by root-to-tip regression analysis, meaning that the location is most compatible with the assumption of a strict molecular clock (correlation = 0.95). The branches are labeled with accession number, host species (African and Asian lineage only), country and collection year of isolation. All isolates in the Brazil lineage were from humans with the exception of 14 USA isolates (KY075938, KY014324, KX838904, KY075939, KY014323, KX838905, KY075937, KY014322, KX838906, KX922708, KY014322, KX838906, KX922708, KY014299) and two Mexican isolates (KX446950, KX446951), which were isolated from Aedes mosquitoes. (DOCX 58 kb) [file 12859_2017_1894_MOESM2_ESM.docx]

**Figure S1**. Maximum likelihood tree of 196 ZIKV complete coding sequences. The tree was rooted by root-to-tip regression analysis, meaning that the location is most compatible with the assumption of a strict molecular clock (correlation = 0.95). The branches are labeled with accession number, host species (African and Asian lineage only), country and collection year of isolation. All isolates in the Brazil lineage were from humans with the exception of 14 USA isolates (KY075938, KY014324, KX838904, KY075939, KY014323, KX838905, KY075937, KY014322, KX838906, KX922708, KY014322, KX838906, KX922708, KY014299) and two Mexican isolates (KX446950, KX446951), which were isolated from *Aedes* mosquitoes.
