# Supplementary material for: Suggested mechanisms for Zika virus causing microcephaly: what do the genomes tell us?
Source: BMC Bioinformatics. 2017 Dec 28;18(Suppl 14):471. doi: 10.1186/s12859-017-1894-3 (PMC5751795; doi:10.1186/s12859-017-1894-3)
Supplement: Supplementary file 3 — K-mer analysis for the three ZIKV lineages. The p-values, calculated by one-sample Wilcoxon test, are given for pairs of lineages. (DOCX 30 kb) [file 12859_2017_1894_MOESM3_ESM.docx]

| **K-mer** | **Number of**  **K-mers analysed** | **Africa:Asia** | **Africa:Brazil** | **Asia:Brazil** |
| --- | --- | --- | --- | --- |
| 1 | 4 | 1.0 | 0.875 | 0.375 |
| 2 | 16 | 0.9321 | 6.104e-05 | 0.0004782 |
| 3 | 64 | 0.9345 | 2.302e-09 | 3.536e-12 |
| 4 | 256 | 0.5439 | 7.66e-15 | < 2.2e-16 |
| 5 | 1024 | 9.707e-07 | 1.184e-05 | < 2.2e-16 |
| 6 | 4044 | 0.6194 | < 2.2e-16 | < 2.2e-16 |
| 7 | 12508 | < 2.2e-16 | < 2.2e-16 | 0.2743 |
| 8 | 23769 | < 2.2e-16 | < 2.2e-16 | < 2.2e-16 |
| 9 | 32428 | < 2.2e-16 | < 2.2e-16 | < 2.2e-16 |
| 10 | 37994 | < 2.2e-16 | < 2.2e-16 | < 2.2e-16 |

**Table S2**. K-mer analysis for the three ZIKV lineages. The p-values, calculated by one-sample Wilcoxon test, are given for pairs of lineages.
