# Supplementary material for: Suggested mechanisms for Zika virus causing microcephaly: what do the genomes tell us?
Source: BMC Bioinformatics. 2017 Dec 28;18(Suppl 14):471. doi: 10.1186/s12859-017-1894-3 (PMC5751795; doi:10.1186/s12859-017-1894-3)
Supplement: Supplementary file 4 — Ratio of observed over expected tetranucleotide frequency in ZIKV genomes in historical isolates up to 2014 (top) and from the French Polynesian outbreak and onwards (bottom). The genomes are ordered according to their position in the phylogenetic tree, with clusters separated by dotted lines. A few individual genomes are listed below the panels for reference. The last two genomes belonging to the Asian lineage 2010–2014 shown in the top panel (far right) are repeated in the lower panel (far left). The tetranucleotide with the highest and lowest frequency in the Brazil lineage are shown by bold orange (TAAT) and red (TATC) lines, respectively. (DOCX 378 kb) [file 12859_2017_1894_MOESM4_ESM.docx]

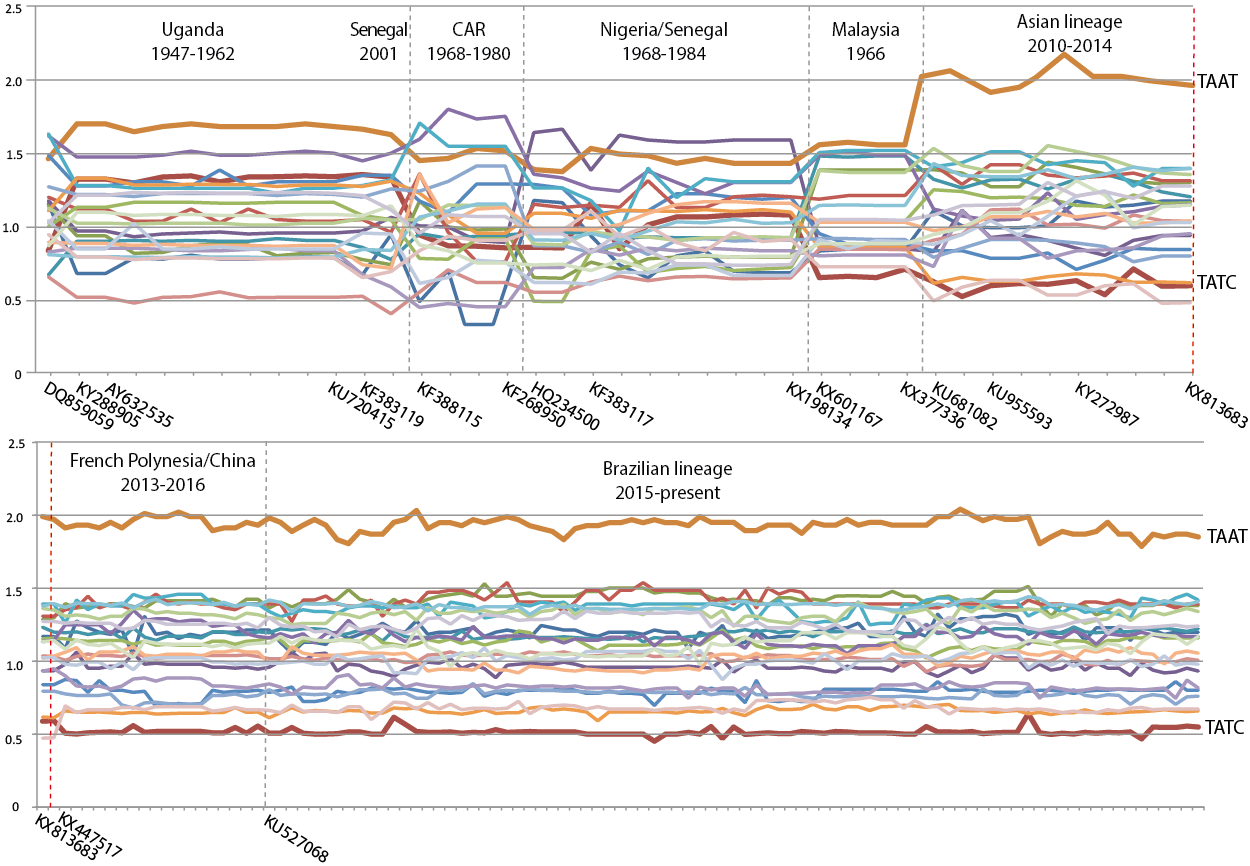


**Figure S2.** Ratio of observed over expected tetranucleotide frequency in ZIKV genomes in historical isolates up to 2014 (top) and from the French Polynesian outbreak and onwards (bottom). The genomes are ordered according to their position in the phylogenetic tree, with clusters separated by dotted lines. A few individual genomes are listed below the panels for reference. The last two genomes belonging to the Asian lineage 2010-2014 shown in the top panel (far right) are repeated in the lower panel (far left). The tetranucleotide with the highest and lowest frequency in the Brazil lineage are shown by bold orange (TAAT) and red (TATC) lines, respectively.
